# Changes in cognitive performance following combined CogSMART-SA and BrainHQ interventions: a pilot study

**DOI:** 10.1186/s12883-026-04778-9

**Published:** 2026-03-21

**Authors:** Hetta Gouse, Zanele Nhlabatsi-Khumalo, Rhiannon Chernotsky, Elizabeth W. Twamley, David Vance, Michelle Henry, Reuben Robbins, Kevin G. F. Thomas

**Affiliations:** 1 Department of Psychiatry & Mental Health, SCID Laboratory, University of Cape Town, Cape Town, South Africa; 2 Department of Psychology, University of New South Wales, Sydney, Australia; 3 ACSENT Laboratory, Department of Psychology, University of Cape Town, Cape Town, South Africa; 4 Department of Psychiatry, Center of Excellence for Stress and Mental Health, University of California, San Diego, CA USA; 5 VA San Diego Healthcare System, San Diego, USA; 6 School of Nursing, University of Alabama at Birmingham, Birmingham, AL USA; 7 Centre for Higher Education Development, University of Cape Town, Cape Town, South Africa; 8 HIV Center for Clinical and Behavioral Studies, Department of Psychiatry, New York State Psychiatric Institute & Columbia University, New York, NY USA; 9 Faculty of Humanities, University of Pretoria, Pretoria, South Africa

**Keywords:** Cognitive Impairment, Cognitive Remediation Therapy, Cognitive Remediation Training, HIV-associated neurocognitive disorders, Rehabilitation, South Africa

## Abstract

**Background:**

Despite effective antiretroviral treatment, neurocognitive impairment (NCI) and functional disability remain concerningly prevalent among people living with HIV (PLWH). Despite this, there are currently no adequate evidence-based treatments targeting NCI in PLWH who reside in low- and middle -income countries. This is a major public health concern given that most the global population of PLWH is concentrated in those countries. Studies emerging from high-income countries of the global north suggest that different forms of cognitive remediation therapy (e.g., compensatory cognitive training [CCT] and restorative computer-based cognitive remediation training [CCRT]) may be helpful in treating NCI in PLWH. This developmental pilot study is the first to culturally adapt and pilot test a CCT approach (CogSMART) and a CCRT approach (BrainHQ©) for use in African PLWH.

**Method:**

Forty-three participants (mean age = 41.1 ± 5.4 years) were randomized into either an intervention group (*n* = 27) or a matched-contact control group (*n* = 16). All participants completed a neuropsychological test battery and self-report measures of mental health and everyday functional competencies at baseline and at study exit. Participants assigned to the intervention group completed 10 CogSMART and 20 BrainHQ© sessions over the 5 weeks between baseline and exit testing.

**Results:**

A significantly smaller proportion of intervention-group participants met criteria for neurocognitive impairment at study exit (30%) than at baseline (70%).

Mixed-model analyses detected a significant and a noteworthy time x group interaction effect for the two cognitive outcomes, Global Deficit Score (p < 0.001) and delayed verbal memory (p = 0.050). In both cases, intervention-group participants made greater cognitive gains than controls participants over the 5-week trial period.

**Conclusions:**

Culturally adapted cognitive remediation therapy shows good potential to reduce symptoms of NCI among South African PLWH. Reduction of such symptoms could, in turn, lead to better quality of life and other functional and health improvements. However, the current results must be replicated using larger-scale randomized controlled trials in the same setting. Future research should also seek to better understand the barriers to, and facilitators of successful implementation of neurorehabilitation interventions for NCI among PLWH in low- and middle-income countries.

**Trial registration:**

ClinicalTrials.gov NCT06466642.

**Supplementary Information:**

The online version contains supplementary material available at 10.1186/s12883-026-04778-9.

## Background

Despite effective antiretroviral treatment (ART), neurocognitive impairment (NCI) remains a common comorbid condition of HIV infection [1, 2]. There are, as yet, no pharmacotherapies that specifically treat NCI in HIV. However, there is evidence that cognitive remediation therapy (CRT) strategies can provide support to PLWH who present with cognitive and functional deficits [3–8].

Persons presenting with HIV-associated NCI typically perform poorly on standardized tests assessing motor speed, information processing speed, attention and concentration, learning, memory, and executive functioning [9–13]. Severity of impairment ranges from asymptomatic to a severe dementia-type form [9, 14], with asymptomatic and mild impairment being most common. Functionally, HIV-associated NCI is associated with a broad range of poorer outcomes including suboptimal ART adherence [15–18], impaired instrumental activities of daily living (e.g., poor planning, driving, and finance management), lower overall quality of life, and increased need for social services [19–25]. Employability is also adversely affected [20, 26].

Cognitive remediation is an intervention aimed at improving cognition (i.e., attention, memory, executive function, social cognition, or metacognition) using scientific principles of learning. The goal is to enhance functional outcomes in everyday life. Cognitive remediation is most effective when it is delivered within a supportive environment (formal or informal) that provides support and opportunity for cognitive gains to real-world situations (see the review by Wykes et al. for an overview of the history of cognitive remediation therapy [27]. Currently, there are two predominant CRT strategies. One is compensatory cognitive training (CCT), a behavioral skills training approach that seeks to help affected people (re)acquire functional skills. CogSMART [28, 29] is a formal evidenced based CCT program [29–32]. A second frequently used CRT strategy is restorative computer-based cognitive remediation training (CCRT). BrainHQ© [33] is an evidenced based computer-based CRT program that aims to improve cognitive functioning by engaging users in repeated, increasingly complex, computer-based game-like activities that cover multiple cognitive domains [30, 34].

The theoretical frameworks guiding CRT programs that were developed in the United States and western Europe (as CogSMART, BrainHQ©, and almost all others were) are also likely to apply in low- and middle-income countries (LMICs). However, without proper adaptation these programs are unlikely to address the socioeconomic, linguistic, and other cultural differences that distinguish the environmental contexts of high-income countries and LMICs [35–37]. Hence, accurate assessment of the efficacy of CRTs in LMICs (e.g., via randomized controlled trial) presupposes that the interventions have been adapted for (a) the neurological or psychiatric condition under scrutiny (e.g., TBI, HIV, schizophrenia), (b) the educational, linguistic, and cultural characteristics of the target population, all while maintaining (c) the core components of CRT [37].

### The current study

For the parent study, we adapted CogSMART [28] and selected culturally relevant BrainHQ© [33] tasks for use with Xhosa-speaking PLWH in South Africa (please refer to the protocol paper for study details [38]). We consulted Xhosa PLWH throughout the adaptation and translation processes of the interventions and examined client perspectives on the interventions. The aim of this pilot study was to assess major outcomes (cognition, mental health, functional abilities) following the interventions. We hypothesized that PLWH who received the intervention will have improved performance on neurocognitive testing, and self-report better mental health and functional outcomes at study exit. Ultimately, we sought to develop the foundation for a solid RCT protocol and to demonstrate, using data from a neuropsychological test battery and a set of self-report measures, that adapted versions of CogSMART and BrainHQ© can potentially be used successfully in a LMIC context to address the gap in care for PLWH with concurrent NCI.

## Methods

### Participants

The original sample of participants comprised 47 PLWH who screened positive for NCI. Due to poor attendance at the intervention sessions by 4 participants in the Control group, the final sample comprised 43 participants (90% retention; female *n* = 36; *M* age = 41.44 ± 5.35 years; Intervention *n* = 27; Control *n* = 16).

Participants were recruited, using convenience and snowball sampling, between April 2023 and March 2024 from two primary healthcare clinics and one non-governmental organization (TBHIV Care) in Cape Town, South Africa. After five participants were recruited, they were allocated to a group that was randomized to the intervention or control condition.

Study inclusion criteria were: 1) age between 30 and 50 years; 2) ≥ 8 years of education; 3) Xhosa home language; 4) HIV-positive; 5) current ART prescription; 6) positive screen for NCI, as indicated by both (a) positive response to at least one question on the HIV Cognitive Symptom Questionnaire (HCSQ [39]) and (b) performing at least one standard deviation below locally established norms on one test from NeuroScreen [40]; 7) ability to understand and sign an informed consent document, as indicated by the University of California San Diego Brief Assessment of Capacity to Consent (UBACC [41]); and 8) willingness to complete two neuropsychological testing sessions (one at study entry and the other at study exit) as well as ten 2-hour CogSMART South Africa (CogSMART-SA [38]) sessions and 10 h (twenty 30-minute sessions) of BrainHQ© activities over the 5 weeks between study entry and exit.

Study exclusion criteria were: (1) a significant neuropsychiatric or neurological comorbidity (e.g., schizophrenia, epilepsy, bipolar disorder, multiple sclerosis, intellectual disability, traumatic brain injury with a loss of consciousness > 30 min), determined via self-report; and (2) medical and other conditions that prevented full study participation (e.g., undergoing radiation, legally blind and/or deaf), determined via self-report.

The University of Cape Town Faculty of Health Sciences Human Research Ethics Committee (HREC REF: 045/2022) gave ethical approval. All study procedures followed ethical guidelines for research involving human subjects outlined in the Declaration of Helsinki [42].

### Procedures

Potential participants were initially screened using a demographic questionnaire, a medical history questionnaire, and the HCSQ [39, 43]. Those eligible to proceed were invited to complete a tablet-based battery of three tests (i.e., NeuroScreen) to determine if they met criteria for NCI [40, 44]. If this criterion was met, they completed the UBACC [41] to confirm capacity to provide informed consent. Once this confirmation was provided the participant signed informed consent and they were formally enrolled into the study. Potential participants who failed to meet capacity to consent to study participation were referred to their treating physician for further investigation.

After five consecutive participants were recruited, they were grouped and block randomized into either the intervention (CogSMART-SA and BrainHQ©) or control arm of the study. Ultimately, the study comprised nine groups (five in the intervention arm). Group sizes ranged between 3 and 5.

Within 14 days of formal enrolment into the study, each participant was administered a comprehensive neuropsychological test battery and a set of self-report measures enquiring about mental health and ability to complete functional activities of daily living. They repeated these assessments within two weeks of competing the interventions, at study exit. Between assessments, they were exposed to either the cognitive remediation interventions or the control procedures. Individual assessment sessions were held at the Neuroscience Institute; intervention or control sessions were held at the TBHIV premises in Khayelitsha, Cape Town. Participants were compensated a total of approximately $266 if they completed all study visits.

#### Cognitive remediation sessions

We adapted the original CogSMART program [29] for use with Xhosa-speaking PLWH in South Africa (Grant Number: 5R34MH126702, protocol paper [38]). The core features of this adapted program, which we refer to as CogSMART-SA, are presented in Table 1. As the table shows, the intervention provided education on the characteristics of HIV-associated NCI and concomitant psychiatric or functional disorders (e.g., depression) and taught compensatory strategies to overcome these cognitive, behavioral, and emotional challenges. Participants attended ten 2-hour group sessions over the 5-week intervention period. Each session was delivered by a lay counsellor.

As a first step, we chose BrainHQ© activities on the profile of cognitive impairment frequently observed in PLWH (e.g., poor performance on tests assessing processing speed and attention, see Table 2 [45–47], and then we chose activities that were culturally fit for our study population. Activity instructions were translated to Xhosa. Participants completed 10 h (twenty 30-minute sessions) of BrainHQ© over the 5-week intervention period. Each session was self-paced and a counsellor was available to assist participants with any technical issues.

**Table 1:** CogSMART-SA: Session Structure, Domains Targeted, and Strategies/Techniques Taught

Session Number	Session Topic(s)	Session Components (Psychoeducation; Compensatory Techniques and Strategies)
1	Introduction to the Course. Introduction to Neurocognitive Disorders.	• Course overview. • Psychoeducation on NCI in PLWH informed by the presentation of HIV-associated Neurocognitive Disorders (HAND) [48].
2	Managing Fatigue, Sleep Problems, and Tension.	• Lifestyle strategies to manage fatigue, sleep problems and tension. • Stress reduction techniques (e.g., progressive muscle relaxation, abdominal breathing, mindfulness, visualization, and grounding). • Muscle relaxation and visualization meditation exercises^a^.
3–4	Organization and Prospective Memory.	• Daily calendar to organize tasks and events. • To-do lists and task prioritization strategies to improve organization. • Linking tasks and “can’t miss reminders” to cue tasks and enhance prospective memory.
5	Attention and Concentration.	• Using conversational vigilance skills to reduce distractions, maintain eye contact, paraphrase, and ask questions. • Developing task vigilance skills by paraphrasing instructions and using self-talk during tasks to maintain focus.
6–7	Learning and Memory.	• Using encoding strategies (e.g., writing things down, paraphrasing, repetition, association, chunking, categorizing, acronyms, rhymes, visual imagery, and name-learning strategies). • Using retrieval strategies (e.g., systematic searching) and organizational strategies for improving learning and memory.
8	Planning and Goal Setting.	• Using the 6-step problem-solving method: define the problem, brainstorm solutions, evaluate solutions, select a solution, try it out, and evaluate effectiveness. • Setting realistic goals and developing plans to achieve them.
9	Problem Solving and Cognitive Flexibility.	• Engaging in self-talk while solving problems to improve cognitive flexibility and adaptability. • Practicing hypothesis testing and self-monitoring to enhance problem-solving skills.
10	Skills Integration, Review, and Next Steps.	• Integrating learned skills into daily life routines and activities. • Reviewing progress and discussing next steps.

*CogSMART-SA* - Cognitive Symptom Management and Rehabilitation Therapy – South African Adaptation

^a^These were translated into Xhosa and were made accessible via a hyperlink where participants could either stream the content or download it to their personal devices

**Table 2:** BrainHQ©: Contents of the South African-Adapted Cognitive Rehabilitation Program

Cognitive Domain	Exercise Name	Targeted Abilities
Attention	1. Fine Tuning	Focus; Visual precision; Handling multiple tasks simultaneously.
	2. Target Tracker	
	3. Double Decision	
	4. Divided Attention	
	5. Mixed Signals	
Processing Speed ^a^	1. Hawk Eye	Visual processing speed; Aauditory processing speed; Noticing fine details.
	2. Eye for Detail	
	3. Sound Sweeps	
	4. Visual Sweeps	

^a^The BrainHQ© software refers to this domain as “Brain Speed”

#### Control procedures

Using matched attention control conditions, the adapted CogSMART-SA control activity consisted of goal-oriented group meetings facilitated by a trained counsellor. Participants chose their own discussion topics.

For BrainHQ© the control activity involved participants playing simple puzzle-based computer games that provide no therapeutic benefits (e.g., Double Klondike Solitaire, Gems Swap, Lineup Four, Brick Breaking Hex, Warship, Reversi).

### Measures

#### Initial screening

A *demographic questionnaire* gathered self-report information on participant age, gender, education, employment, income, and type of housing. A 24-item *medical history questionnaire* gathered self-report information on participant health, including years since HIV diagnosis and years taking ART. The 5-item *HIV Cognitive Symptom Questionnaire (HCSQ)* gathered self-reported data on cognitive symptoms [39]. NeuroScreen was used as an objective screen for cognitive symptoms [38, 40].

#### Outcome measures

In this secondary analysis we addressed cognitive test performance and functional outcomes following the interventions. Cognition was assessed using a comprehensive neuropsychology battery using the Global Deficit Score [49].

#### Neuropsychological test battery

This comprehensive battery was administered by a trained neuropsychology technician under the supervision of a licensed clinical neuropsychologist. The battery assessed performance across six cognitive domains: (1) *motor function*: Grooved Pegboard Test (GPT) – outcome variables completion time for dominant hand non-dominant hand respectively; (2) *processing speed*: Symbol Search (Wechsler Adult Intelligence Scale-Third Edition (WAIS-III [50])), Coding subtests (WAIS-III), and the Color Trails Test 1 (CTT1) – the outcome variable for the latter was time to completion; (3) *attention*: Digit Span (WAIS-III) – Total score; (4) *verbal memory*: Hopkins Verbal Learning Test (HVLT) – outcome variables were total number of words correctly remembered across the three immediate recall trials (learning), and total number of words correctly remembered on the delayed recall trial; (5) *verbal fluency*: Category Fluency Test – outcome variables were the total number of animals and total number of fruits and vegetables generated within 60 s; and (6) *executive functioning*: Color Trails Test 2 (CTT 2) – outcome variable was time to completion. This assessment required approximately 2.5 h to complete.

#### Measures of mental health and everyday functional competencies

We used a selection of self-report measures to gather information related to ART adherence, mental well-being, and cognitive and social outcomes, all of which had previously been used in South African HIV research studies and have, at least, adequate psychometric properties suited to the purposes for which they were used here.

A 5-item *medication adherence questionnaire* [16, 17] assessed ART adherence. Participants responded to each item using a Likert-type scale, with anchors at 0 (*Always True*) and 5 (*Never True*). Higher scores indicate better adherence.

The 20-item *Center for Epidemiologic Studies-Depression* (*CES-D*) scale [51] assessed for the presence of depressive symptoms (e.g., feelings of sadness, hopelessness, worthlessness, loss of interest or pleasure in activities). We used the standard cut-off score of 16; scores higher than that suggest the respondent reports significant depressive experiences.

The 11-item *Patient-Reported Outcomes Measurement Information System* (*PROMIS*) [52] scale assessed overall health difficulties. Participants responded to each item using a Likert-type scale, with anchors at 1 (*Poor*) and 5 (*Excellent*). Higher scores indicate better overall health.

The 7-item *HIV Social Outcomes Questionnaire* (*HSOQ*) assessed for the presence of social withdrawal. Participants responded to each item using a binary scale (0 = *No*, 1 = *Yes*). Higher scores indicate more symptoms of social withdrawal.

The 8-item *Applied Cognition General Concerns Scale (ACGC)* [52] assessed for the presence of general concerns over the 7 days prior to reporting. Participants responded to each item using a Likert-type scale, with anchors at 1 (*Never)* and 5 (*Very often*). Higher scores indicate the presence of more such concerns.

The 60-item *Cognitive Problems and Strategies Assessment (CPSA) scale* [53] assessed self-reported cognitive problems and cognitive strategies by asking questions such as “I have difficulty remembering to do things that I have scheduled” and “I keep a written list of things I need to do”. Participants responded to each item indicating how often a certain problem with memory or thinking arose or how often they use a particular type of memory or problem-soling strategy to avoid or address such problems. Response was required on a Likert-type scale, with anchors at 0 (*Rarely/Never*) and 3 (*Always*). Higher scores indicate more cognitive problems and little by way of strategy employment.

The 8-item *Satisfaction with Social Roles and Activities* scale [54] assessed for the presence of distress related to social roles as well as participant satisfaction with social roles and activities over the 7 days prior to reporting. Participants responded to each item on a Likert-type scale with anchors at 0 (*Never*) and 4 (*Always*). Higher scores indicate greater satisfaction with social roles and activities.

### Data management and statistical analyses

We analyzed data collected at baseline and at study exit. The analyses were completed using R version 1.2 (R Foundation for Statistical Computing, Austria), with the threshold for statistical significance set at *p* < 0.05 and effect sizes interpreted following conventional guidelines [55].

The analyses proceeded across five discrete steps. First, we compiled a complete set of descriptive statistics to summarize the sample’s sociodemographic and clinical characteristics and then used *t*-tests or Fisher’s exact tests to compare those characteristics across the Intervention and Control groups.

Second, we used independent samples *t*-tests or Mann-Whitney *U* tests (for continuous variables) and chi-square tests or Fisher’s exact tests (for categorical variables) to conduct between-group comparisons for all major outcomes (i.e., performance on neuropsychological tests and self-reports on measures of mental health and everyday functional competencies) at baseline.

Third, our primary set of analyses evaluated the effects of the interventions directly. Here, we introduced an important outcome variable estimating overall cognitive performance: the Global Deficit Score (GDS). To calculate the GDS, we first standardized raw scores to *z*-scores. Then, for each participant (a) each *z*-score was converted to a *T*-score using the formula *T* = 10*z* + 50; (b) each *T*-score was converted into a deficit score; and (c) the deficit scores were averaged to obtain the GDS [49]. Participants with GDS scores > 0.5 were classified as presenting with NCI. Specifically, linear mixed effects models (LMM) evaluated the impact of exposure to CogSMART-SA and BrainHQ© interventions on neuropsychological test performance, self-reported mental health, and self-reported everyday functional competencies. Details of the models were as follows: (a) fixed effects were Group (Intervention versus control) and Time (assessment at baseline versus assessment at study exit), (b) a random effect was participant ID, included to account for repeated measures, and (c) the interaction term was Group x Time, included to assess differential impact of the intervention versus control exposure over the period of evaluation. Furthermore, each model was adjusted for age (the only sociodemographic or clinical variable on which prior analyses had detected a statistically significant between-group difference), and each model’s outcome variable was a change score (viz., the difference between the value of the variable at baseline and that at study exit).

We followed up the LMM with GDS as outcome variable with two secondary analyses: (a) dependent sample *t*-tests compared GDS scores at baseline and study exit, within the Intervention and Control groups separately, and (b) a McNemar Chi-square test of contingency compared the proportion of participants in each group presenting with NCI (as determined by standard GDS criteria) at baseline and study exit.

## Results

### Sample sociodemographic and clinical characteristics

As Table 3 shows, the groups were well matched on almost all these variables. However, on average participants assigned to the Intervention group were statistically significantly younger than those assigned to the Control group (40.52 vs. 43.00 years old). For the overall study sample, mean age was 41.44 ± 5.35 years, mean years since HIV diagnosis was 13.45 ± 6.50, and mean years on ART was 11.28 ± 5.49.

**Table 3:** Sample Sociodemographic and Clinical Characteristics (*N* = 43)

	Group						
Variable	Intervention (*n* = 27)		Control (*n* = 16)				
	M (SD)	f (%)	M (SD)	f (%)	Test Statistic^a^	*p*	ESE
Age (years)	40.52 (5.57)		43.00 (4.72)		2.22	0.030*	-0.47
Education (years completed)	10.26 (1.23)		9.94 (1.29)		-1.02	0.311	0.23
Sex						1.000	
Male		4 (14.81)		3 (18.75)			
Female		23 (85.19)		13 (81.25)			
Employment Status						1.000	
Unemployed		23 (85.19)		14 (87.5)			
Employed		14 (14.81)		2 (12.50)			
Income						0.164	
ZAR0–R1500		7 (25.93)		8 (50.00)			
ZAR1501–R5000		18 (66.67)		6 (37.5)			
> ZAR5000		2 (7.41)		2 (12.5)			
Housing Type					0.03	0.845	
Shack / wendy house / backyard dwelling		19 (70.37)		10 (62.5)			
Own / family house		8 (29.63)		6 (37.5)			
CES-D Total Score	28.15 (9.36)		30.75 (11.05)		-0.82	0.415	-0.26
Years since HIV diagnosis	14.03 (6.44)		11.98 (6.52)		-1.14	0.260	0.31
Years on ART	11.68 (5.54)		10.00 (5.41)		-1.11	0.272	0.30

*M *Mean, *SD*  Standard deviation, *f*  Frequency of occurrence within the sample, *ZAR* South African rands, *CES-D* Center for Epidemiologic Studies-Depression scale, *ART* Antiretroviral treatment, *ESE* Effect size estimate (in this case, Cohen’s *d* for continuous variables)

^a^For continuous variables(Age, Education, CES-D Total Score, Years since HIV diagnosis, Years on ART), the test statistic was t; for most categorical variables (Sex, Employment Status, and Income), the test statistic was Fisher’s exact test; for the categorical variable Housing Type, the test statistic was χ^2^

*p < .05

### Between-group comparisons: neuropsychological test performance, mental health, and everyday functional competencies at baseline

Analyses detected no significant between-group differences regarding (a) baseline performance on the set of standardized neuropsychological tests, and (b) baseline self-reports on the measures of mental health and everyday functional competencies (see Additional File, Tables AF1–AF4).

### Primary analysis: direct evaluation of the intervention effects

The model evaluating the impact of the interventions on overall cognitive performance detected a significant Group x Time interaction effect (*p* < 0.001; see Table 4). The direction of this finding (based on observations of the relevant descriptive statistics; see Additional File, Table AF1) suggests there was a substantially greater change in GDS among Intervention-group participants than among Control-group participants, with the direction of change indicating an improvement in cognitive performance from baseline to study exit.

**Table 4:** Linear Mixed Model Results for Global Deficit Score (GDS) Outcome Variable (*N* = 42)

Global Deficit Score (GDS)									
Model Fit	ICC	*R*^2^_c							
	0.885	0.894							
						95% CI			
Fixed Effects			Estimate	*SE*	*t*	LL	UL	*p*	ESE
Intercept			0.53	0.65	0.80	-0.47	0.49	0.427	0.24
Main effect: Group			0.17	0.17	0.97	-0.31	0.91	0.337	0.31
Main effect: Time			-0.05	0.05	1.15	-0.07	0.26	0.252	0.08
Covariate: Age			0.00	0.01	0.14	-0.25	0.29	0.887	0.14
Interaction effect: Group x Time			-0.45	0.06	-7.47	-1.00	-0.58	< 0.001***	0.11
Random Effects	Variance	*SD*							
Intercept (pid)	0.28	0.53							
Residual	0.04	0.19							

This model was conducted with a sample of data from 42 participants (Intervention group *n* = 26, Control group *n* = 16 – the data from one participant in the Intervention group were excluded due to extreme outlying values)

*ICC* Interclass correlation coefficient, *R*^2^_c Conditional *R*^2^, *SE* Standard error of the estimate, *95% CI* 95% confidence interval, *LL* Lower limit, *UL* Upper limit, *ESE* Effect size estimate (Cohen’s *f*), *pid* Participant identifier, *SD *Standard deviation

***p < 0.001

We explored what might be driving the significant GDS effect. Among the models evaluating the impact of the interventions on individual neuropsychological test performance, only one detected a close-to-significant Group x Time interaction effect: For delayed verbal memory (HVLT delayed recall), *p* = 0.050, with a moderate effect size of Cohen’s *f* = 0.55 (see Table 5). The direction of this finding (again, based on observations of the relevant descriptive statistics; see Additional File, Table AF1) suggests there was a substantially greater change in delayed verbal recall among Intervention-group participants than among controls, with the direction of change indicating an improvement in performance from baseline to study exit.

**Fig. 1:**
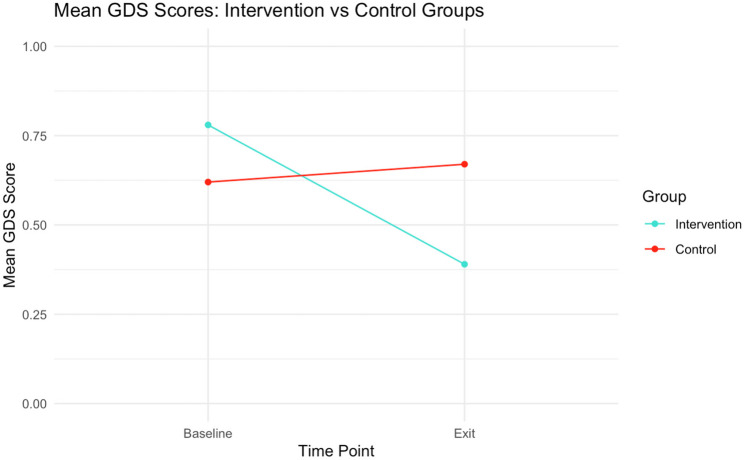
Mean GDS in the Intervention and Control Groups: Comparison Over Time (*N* = 43) Note. The figure shows a comparison of how overall cognitive performance changed for the two groups from baseline (first neuropsychological assessment, at study entry) to study exit (second neuropsychological assessment). GDS = Global Deficit Score. Sample sizes: Intervention group *n* = 26, Control group *n* = 17

Further regarding the effects of the intervention on overall cognitive performance, Fig. 1 shows GDS scores at baseline and at study exit for the Intervention and Control groups. The mean GDS for the Intervention group was significantly lower at study exit compared to baseline (0.30 vs. 0.78; *p* = 0.001, Cohen’s *d* = 1.37), suggesting a marked improvement in overall cognitive performance across the intervention period. In contrast, the mean GDS for the Control group remained relatively stable from baseline to study exit (0.62 vs. 0.67; *p* = 0.485, Cohen’s *d* = 0.18).

**Fig. 2:**
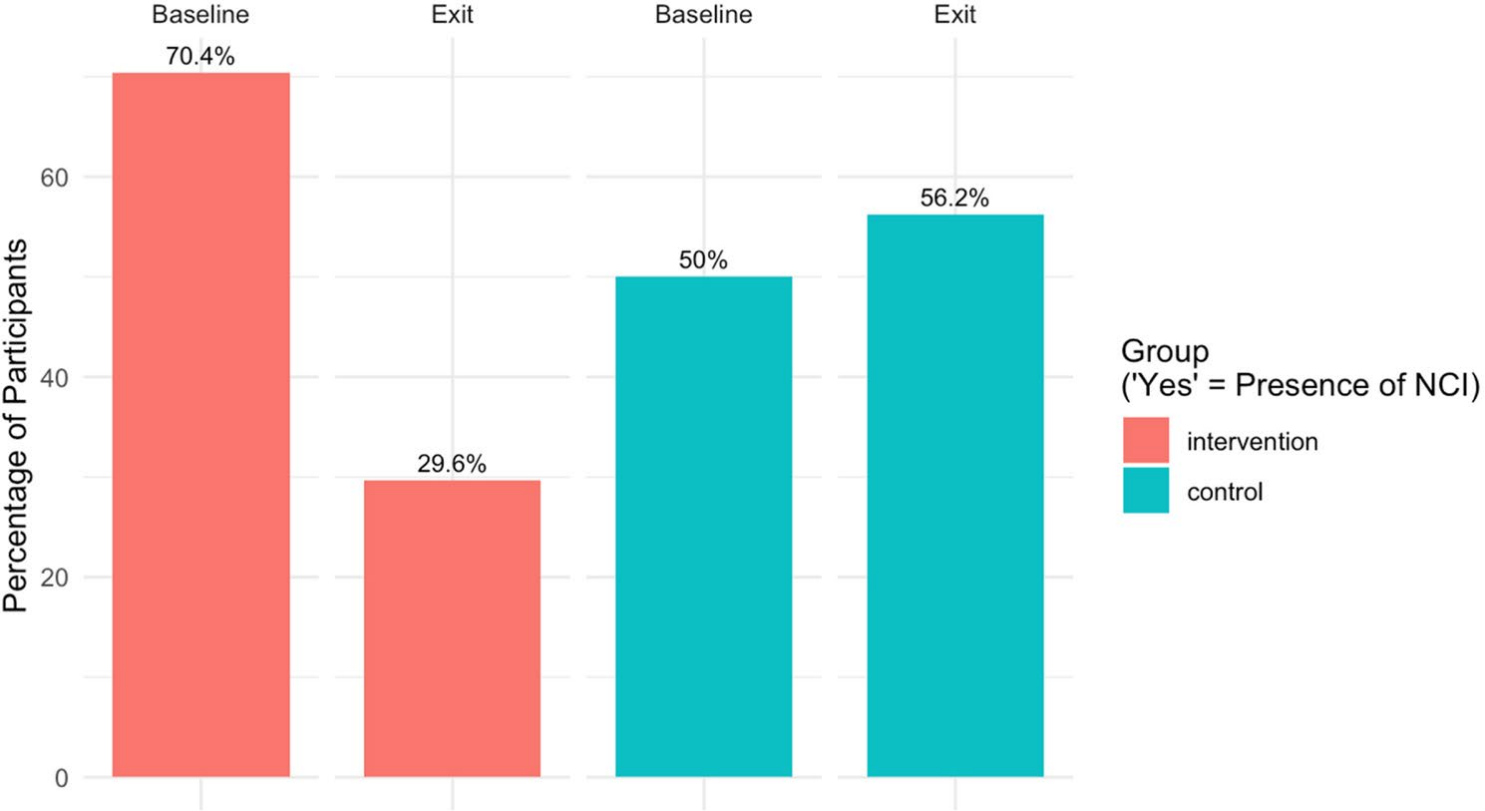
Proportion of Participants in the Intervention and Control groups Meeting Criteria for NCI: Comparison Over Time (*N* = 43 Note. The figure shows a comparison of how the proportion of participants in each group whomet standard GDS-based criteria for NCI changed from baseline (first neuropsychologicalassessment, at study entry) to study exit (second neuropsychological assessment).GDS = Global Deficit Score; NCI = neurocognitive impairment. Sample sizes: Intervention group *n* = 26, Control group *n* = 17

Figure 2 shows, for each group separately, the proportion of participants presenting with NCI at baseline and at study exit. Within the Intervention group, there was a substantial reduction in this proportion over time (70.37% vs. 29.63%; McNemar test *p* = 0.003), whereas the proportions within the Control group remained relatively stable (50.00% vs. 56.25%; McNemar test *p* = 1.00).

**Table 5:** Linear Mixed Model Results for Hopkins Verbal Learning Test (HVLT) Delayed Recall Outcome Variable (*N* = 43)

HVLT Delayed Recall									
Model Fit	ICC	*R*^2^_c							
	0.61	0.65							
						95% CI			
Fixed Effects			Estimate	*SE*	*t*	LL	UL	*p*	ESE
Intercept			-3.12	1.09	-2.85	-0.43	-1.55	0.007**	0.06
Main effect: Group			0.21	0.30	0.72	-0.40	0.85	0.474	0.23
Main effect: Time			-0.05	0.20	-0.27	-0.48	0.37	0.792	-0.06
Covariate: Age			0.04	0.02	1.77	0.03	0.52	0.085	0.24
Interaction effect: Group x Time			-0.52	0.26	-2.02	-1.09	-0.01	0.050	-0.55
Random Effects	Variance	SD							
Intercept (pid)	0.51	0.72							
Residual	0.33	0.58							

This model was conducted using *z*-score data from the Hopkins Verbal Learning Test (HVLT) delayed recall trial, which is an assessment of delayed verbal memory. Sample sizes: Intervention group *n* = 26, Control group *n* = 17

*ICC* Interclass correlation coefficient, *R*^*2*^_c Conditional *R*^2^, *SE* Standard error of the estimate, *95% CI* 95% confidence interval, *LL* Lower limit, *UL* Upper limit, *ESE* Effect size estimate (Cohen’s *f*), *pid* Participant identifier, *SD *Standard deviation

**p < 0.01

Among the models evaluating the impact of the interventions on measures of mental health and everyday functional competencies, analyses detected no significant Group x Time interaction effects.

## Discussion

The current study used data from a parent protocol that sought to adapt two existing cognitive remediation training programs (CogSMART [28, 29] and BrainHQ© [33]) for use in South African samples of people living with HIV. The adaptation incorporated changes to the source materials in order to make it suitable for PLWH as well as culturally and linguistically appropriate for use in Black African Xhosa-speaking individuals [38]. The primary aim of this study was to analyze outcome data from a pilot study (*N* = 43 PLWH; 27 assigned to the intervention condition and 16 assigned to a control condition) that implemented both adapted CRT programs over a 5-week period. We thereby hoped to gauge whether the adapted interventions show promise of efficacy for treating PLWH with NCI and hence provide support for a large-scale randomized controlled trial. Our main findings were that exposure to the interventions (but not the control condition) appeared to have a significant positive effect on cognitive performance, and that self-reported mental health and everyday functional competence improved in both the intervention and control groups.

Most participants in the current sample had been living with HIV for about 10 years. All were of low socio-economic status, with most (> 85%) being unemployed. Approximately two-thirds lived in makeshift dwellings as opposed to formal structures owned by themselves or their families. Hence, the interventions were implemented in a sample of PLWH with low levels of financial and health resources and that is in urgent need of support.

Although this was a small and statistically underpowered pilot study, we highlight three particular findings indicating that CogSMART-SA and BrainHQ© should, at least, be the subject of large-scale RCTs in South African PLWH. First, a comparison of baseline and study exit GDS data showed that participants assigned to the Intervention group (but not those assigned to the Control group) showed significant improvement in overall cognitive performance over the intervention period (*p* = 0.001). Second, the proportion of Intervention-group participants who met standard criteria for NCI dropped significantly from baseline to study exit (70% to 30%, *p* = 0.003); this proportion remained relatively stable among those assigned to the Control group. Third, analyses detected a near significant (*p* = 0.05) Group x Time intervention effect for the neuropsychological test measuring delayed verbal memory - on average, participants assigned to the Intervention group showed significant improvement from baseline to study exit, whereas those assigned to the Control group did not. Together, these three findings suggest that exposure to these CRT programs had a positive effect on global cognitive performance in this sample of South African PLWH. Although it is perhaps concerning that the statistical significance of these positive effects was observed on only one of the individual neuropsychological tests, it is notable that the test in question assessed a domain of functioning (memory) that is addressed directly and consistently by the CogSMART-SA sessions.

For other individual neuropsychological tests, as well as for self-reported mental health and everyday functional competence, our analyses did not detect significant Group x Time interaction effects (i.e., specific effects of exposure to the interventions over time). One way to account for these findings is that the social interaction participants (in both the Intervention and Control groups) experienced in a safe, non-stigmatizing space improved their overall sense of wellbeing. This notion is supported by qualitative reports—participants indicated that they found exceptional value in the group experience, that they formed strong relationships with others in their cohort, and that the support they found during study sessions persisted into their everyday lives. We document more of those reports in an upcoming manuscript.

### Limitations and directions for future research

We identify three major limitations that constrain the inferences we might draw from the current observations.

First, and most importantly, this was a pilot study featuring a relatively small sample. Hence, our analyses lacked statistical power to detect some of the effects of interest. Nonetheless, the promising trends we identified offer evidence for the feasibility and possible efficacy of these CRTs in South African (and other LMIC) settings. We argue that, on these bases, a large-scale RCT is justified.

Second, our study sample contained only 7 men (approximately 16% of the overall *N*). Hence, we could not explore gender effects within the data, and we cannot comment on whether the findings generalize broadly across gender groups. In mitigation of the sample’s gender bias is the fact that, approximately 60% of PLWH in South Africa are women [56]. Furthermore, South African men living with HIV are less inclined to access the healthcare system. Future studies should make directed efforts to recruit more men into their samples.

Third, participant use of BrainHQ© was limited by availability of resources and space for this study and participants only completed 10 h of BrainHQ© activities. This is the minimum number of hours generally recommended for these activities, and it may not have been adequate to impart a true effect. Future studies should allow for additional BrainHQ© sessions.

Fourth, because participants were exposed to the CogSMART-SA and BrainHQ© interventions simultaneously, it is not possible to tease apart the distinct effects of each on the measured outcomes. The large-scale RCTs proposed earlier may be able to answer this question by assigning separate groups of participants to three treatment arms: CogSMART-SA, BrainHQ©, and CogSMART-SA+ BrainHQ©.

## Conclusion

PLWH with co-occurring neurocognitive impairment face a significant and troubling gap in care. The overall aim of the parent study within which the current sub-study is nested was to address this gap by piloting cognitive remediation training programs that can augment current treatment. Our findings suggest that a combination of behavioral and computer-based cognitive remediation training interventions improves overall cognitive performance in South African Xhosa-speaking PLWH.

This promising finding on an objective measure of cognitive functioning suggests there is a positive effect of the intervention. Ultimately, improved cognitive functioning can have positive knock-on effects on activities of daily living (e.g., medication adherence, self-care, and the ability to work and care for your family). This developmental study paves the way for a larger RCT that can assess the validity of the adapted CogSMART-SA and Brain HQ© interventions, as well as for a real-world implementation study.

## Supplementary Information

Supplementary Material 1.

## Data Availability

The datasets used and/or analysed during the current study are available from the corresponding author on reasonable request.

## References

[1] Zenebe Y, Necho M, Yimam W, Akele B. Worldwide Occurrence of HIV-Associated Neurocognitive Disorders and Its Associated Factors: A Systematic Review and Meta-Analysis. Front Psychiatry. 2022;13:814362. 35711575 10.3389/fpsyt.2022.814362 PMC9193596

[2] Mekuriaw B, Belayneh Z, Teshome W, Akalu Y. Prevalence and variability of HIV/AIDS-associated neurocognitive impairments in Africa: a systematic review and meta-analysis. BMC Public Health. 2023;23(1):997. 37254121 10.1186/s12889-023-15935-x PMC10228136

[3] Livelli A, Orofino GC, Calcagno A, Farenga M, Penoncelli D, Guastavigna M, et al. Evaluation of a Cognitive Rehabilitation Protocol in HIV Patients with Associated Neurocognitive Disorders: Efficacy and Stability Over Time. Front Behav Neurosci. 2015;9:306. 26635558 10.3389/fnbeh.2015.00306 PMC4644912

[4] Mayo NE, Levine B, Brouillette MJ, Bélanger D, Fellows LK. Efficacy potential of Goal Management Training to improve cognitive function in older people living with HIV. Contemp Clin Trials Commun. 2022;30:101023. 36345346 10.1016/j.conctc.2022.101023 PMC9636436

[5] Fischer EL, Renaud A, Grivaz P, Di Liberto G, Ryvlin P, Cavassini M, et al. Advances in assessment and cognitive neurorehabilitation of HIV-related neurocognitive impairment. Brain Commun. 2025;7(1):fcae399. 39726816 10.1093/braincomms/fcae399 PMC11670355

[6] Zondo S. The cognitive remediation of attention in HIV-associated neurocognitive disorders (HAND): A meta-analysis and systematic review. F1000Res. 2023;12:1133. 38778812 10.12688/f1000research.132166.1 PMC11109681

[7] Eaton AD, Craig SL, Rourke SB, Sota T, Mccullagh JW, Fallon BA, et al. Cognitive remediation group therapy compared to mutual aid group therapy for people aging with HIV-associated neurocognitive disorder: randomized, controlled trial. Social Work Groups. 2022;45(2):116–31. 10.1136/bmjopen-2019-033183 PMC6830703 31676660

[8] Eaton AD, Hui J, Muchenje M, Kon T, Murzin K, Chan Carusone S, et al. Adapting Cognitive Remediation Group Therapy Online: Focus Groups with People Aging with HIV. J Int Association Providers AIDS Care. 2024;23:23259582241242703. 10.1177/23259582241242703 PMC10976509 38545687

[9] Grant H, Masliah, Ellis, Marcotte MC, et al. Neurocognitive disturbances in HIV, including HIV-associated dementia. Biol Psychiatry. 1997;42(1):0.

[10] Heaton RK, Clifford DB, Franklin DR Jr., Woods SP, Ake C, Vaida F, et al. HIV-associated neurocognitive disorders persist in the era of potent antiretroviral therapy: CHARTER Study. Neurology. 2010;75(23):2087–96. 21135382 10.1212/WNL.0b013e318200d727 PMC2995535

[11] Heaton RK, Franklin DR Jr., Deutsch R, Letendre S, Ellis RJ, Casaletto K, et al. Neurocognitive change in the era of HIV combination antiretroviral therapy: the longitudinal CHARTER study. Clin Infect Dis. 2015;60(3):473–80. 25362201 10.1093/cid/ciu862 PMC4303775

[12] Sacktor N, McDermott MP, Marder K, Schifitto G, Selnes OA, McArthur JC, et al. HIV-associated cognitive impairment before and after the advent of combination therapy. J Neurovirol. 2002;8(2):136–42. 11935465 10.1080/13550280290049615

[13] Sacktor N, Skolasky R, Selnes OA, Watters M, Poff P, Shiramizu B, et al. Neuropsychological test profile differences between young and old human immunodeficiency virus-positive individuals. J Neurovirol. 2007;13(3):203–9. 17613710 10.1080/13550280701258423

[14] Antinori A, Arendt G, Becker JT, Brew BJ, Byrd DA, Cherner M, et al. Updated research nosology for HIV-associated neurocognitive disorders. Neurology. 2007;69(18):1789–99. 17914061 10.1212/01.WNL.0000287431.88658.8b PMC4472366

[15] Barclay TR, Hinkin CH, Castellon SA, Mason KI, Reinhard MJ, Marion SD, et al. Age-associated predictors of medication adherence in HIV-positive adults: Health beliefs, self-efficacy, and neurocognitive status. Health Psychol. 2007;26(1):40. 17209696 10.1037/0278-6133.26.1.40 PMC2863998

[16] Ettenhofer ML, Hinkin CH, Castellon SA, Durvasula R, Ullman J, Lam M, et al. Aging, neurocognition, and medication adherence in HIV infection. Am J Geriatr Psychiatry. 2009;17(4):281–90. 19307857 10.1097/JGP.0b013e31819431bd PMC2679810

[17] Hinkin CH, Castellon SA, Durvasula RS, Hardy DJ, Lam MN, Mason KI, et al. Medication adherence among HIV+ adults - Effects of cognitive dysfunction and regimen complexity. Neurology. 2002;59(12):1944–50. 12499488 10.1212/01.wnl.0000038347.48137.67 PMC2871670

[18] Waldrop-Valverde D, Ownby RL, Wilkie FL, Mack A, Kumar M, Metsch L. Neurocognitive aspects of medication adherence in HIV-positive injecting drug users. AIDS Behav. 2006;10(3):287–97. 16485072 10.1007/s10461-005-9062-6

[19] Heaton RK, Franklin DR, Ellis RJ, McCutchan JA, Letendre SL, LeBlanc S, et al. HIV-associated neurocognitive disorders before and during the era of combination antiretroviral therapy: differences in rates, nature, and predictors. J Neurovirol. 2011;17(1):3–16. 21174240 10.1007/s13365-010-0006-1 PMC3032197

[20] Heaton RK, Marcotte TD, White DA, Ross D, Meredith K, Taylot MJ, et al. Nature and vocational significance of neuropsychological impairment associated with HIV infection. Clin Neuropsychol. 1996;10(1):1–14.

[21] Gorman AA, Foley JM, Ettenhofer ML, Hinkin CH, van Gorp WG. Functional consequences of HIV-associated neuropsychological impairment. Neuropsychol Rev. 2009;19(2):186–203. 19472057 10.1007/s11065-009-9095-0 PMC2871666

[22] Woods SP, Iudicello JE, Moran LM, Carey CL, Dawson MS, Grant I, et al. HIV-associated prospective memory impairment increases risk of dependence in everyday functioning. Neuropsychology. 2008;22(1):110–7. 18211160 10.1037/0894-4105.22.1.110 PMC2249562

[23] Woods SP, Iudicello JE, Morgan EE, Verduzco M, Smith TV, Cushman C, et al. Household Everyday Functioning in the Internet Age: Online Shopping and Banking Skills Are Affected in HIV-Associated Neurocognitive Disorders. J Int Neuropsychol Soc. 2017;23(7):605–15. 28625210 10.1017/S1355617717000431 PMC5703204

[24] Ranka JL, Chapparo CJ. Assessment of productivity performance in men with HIV Associated Neurocognitive Disorder (HAND). Work. 2010;36(2):193–206. 20634613 10.3233/WOR-2010-1020

[25] Thomas R, Friebel R, Barker K, Mwenge L, Kanema S, Vanqa N, et al. Work and home productivity of people living with HIV in Zambia and South Africa. AIDS. 2019;33(6):1063–71. 30946160 10.1097/QAD.0000000000002160 PMC6467557

[26] Albert SM, Marder K, Dooneief G, Bell K, Sano M, Todak G, et al. Neuropsychologic impairment in early HIV infection. A risk factor for work disability. Arch Neurol. 1995;52(5):525–30. 7733849 10.1001/archneur.1995.00540290115027

[27] Wykes T, Bowie CR, Cella M. Thinking About the Future of Cognitive Remediation Therapy Revisited: What Is Left to Solve Before Patients Have Access? Schizophr Bull. 2024;50(5):993–1005. 38780191 10.1093/schbul/sbae075 PMC11349022

[28] Twamley EW. Cognitive Symptom Management and Rehabilitation Therapy. San Diego: CogSMART; 2025 [cited 2025 March 3]. Available from: http://www.cogsmart.com/.

[29] Twamley EW, Thomas KR, Gregory AM, Jak AJ, Bondi MW, Delis DC, et al. CogSMART Compensatory Cognitive Training for Traumatic Brain Injury: Effects Over 1 Year. J Head Trauma Rehabil. 2015;30(6):391–401. 25033034 10.1097/HTR.0000000000000076

[30] Attarha M, Mahncke H, Merzenich M. The Real-World Usability, Feasibility, and Performance Distributions of Deploying a Digital Toolbox of Computerized Assessments to Remotely Evaluate Brain Health: Development and Usability Study. JMIR Form Res. 2024;8:e53623. 38739916 10.2196/53623 PMC11130778

[31] Howe EI, Fure SCR, Løvstad M, Enehaug H, Sagstad K, Hellstrøm T, et al. Effectiveness of Combining Compensatory Cognitive Training and Vocational Intervention vs. Treatment as Usual on Return to Work Following Mild-to-Moderate Traumatic Brain Injury: Interim Analysis at 3 and 6 Month Follow-Up. Front Neurol. 2020;11:1414. 10.3389/fneur.2020.561400 PMC7683428 33240196

[32] Howe EI, Løvstad M, Lango KS, Hellstrøm T, Spelkavik Ø, Ugelstad H, et al. Feasibility of a cognitive rehabilitation program for individuals with mild-to moderate traumatic brain injury: Participants’ engagement and satisfaction. Cogent Med. 2019;6(1):1-16.

[33] Posit Science. BrainHQ from Posit Science [Internet]. 2025 [cited 2025 Jan 30]. Available from: https://www.brainhq.com/?v4=true&fr=y.

[34] Vance DE, Fazeli PL, Cheatwood J, Nicholson WC, Morrison SA, Moneyham LD. Computerized Cognitive Training for the Neurocognitive Complications of HIV Infection: A Systematic Review. J Assoc Nurse Aids C. 2019;30(1):51–72. 10.1097/JNC.0000000000000030 30586083

[35] Sweetland AC, Oquendo MA, Sidat M, Santos PF, Vermund SH, Duarte CS, et al. Closing the mental health gap in low-income settings by building research capacity: perspectives from Mozambique. Ann Glob Health. 2014;80(2):126–33. 24976551 10.1016/j.aogh.2014.04.014 PMC4109687

[36] Mellins CA, Nestadt D, Bhana A, Petersen I, Abrams EJ, Alicea S, et al. Adapting Evidence-Based Interventions to Meet the Needs of Adolescents Growing Up with HIV in South Africa: The VUKA Case Example. Global Social Welf. 2014;1(3):97–110. 10.1007/s40609-014-0023-8 PMC4431642 25984440

[37] Dark FL, Amado I, Erlich MD, Ikezawa S. International Experience of Implementing Cognitive Remediation for People With Psychotic Disorders. Schizophr Bull. 2024;50(5):1017–27. 38758086 10.1093/schbul/sbae071 PMC11349011

[38] Nhlabatsi ZC, Robbins R, Twamley R, Vance E, Booyens D, Thomas L, Gouse KH. Cognitive remediation training for people living with HIV in a low resource setting: A developmental study protocol. JMIR Preprints. 01/06/. 10.2196/preprints.78123.

[39] Gouse H, Casson-Crook M, Decloedt EH, Joska JA, Thomas KGF. Adding a brief self-report cognitive tool to the IHDS improves effectiveness of identifying patients with HIV-associated dementia in South Africa. J Neurovirol. 2017;23:686-95. 10.1007/s13365-017-0551-y 28748448

[40] Robbins RN, Gouse H, Brown HG, Ehlers A, Scott TM, Leu CS, et al. A Mobile App to Screen for Neurocognitive Impairment: Preliminary Validation of NeuroScreen Among HIV-Infected South African Adults. JMIR Mhealth Uhealth. 2018;6(1):e5. 29305338 10.2196/mhealth.9148 PMC5775487

[41] Jeste DV, Palmer BW, Appelbaum PS, Golshan S, Glorioso D, Dunn LB, et al. A new brief instrument for assessing decisional capacity for clinical research. Arch Gen Psychiatry. 2007;64(8):966–74. 17679641 10.1001/archpsyc.64.8.966

[42] World Medical Association Declaration. of Helsinki: ethical principles for medical research involving human subjects. JAMA. 2013;310(20):2191–4. 24141714 10.1001/jama.2013.281053

[43] Nyamayaro P, Chibanda D, Robbins RN, Hakim J, Gouse H. Assessment of neurocognitive deficits in people living with HIV in Sub Saharan Africa: A systematic review. Clin Neuropsychol. 2019;33(sup1):1-26. 10.1080/13854046.2019.1606284 PMC7758090 31043112

[44] Robbins RN, Brown H, Ehlers A, Joska JA, Thomas KG, Burgess R, et al. A Smartphone App to Screen for HIV-Related Neurocognitive Impairment. J Mob Technol Med. 2014;3(1):23–6. 24860624 10.7309/jmtm.3.1.5 PMC4029593

[45] Vance DE, Fazeli PL, Ross LA, Wadley VG, Ball KK. Speed of processing training with middle-age and older adults with HIV: a pilot study. J Association Nurses AIDS Care: JANAC. 2012;23(6):500–10. 22579081 10.1016/j.jana.2012.01.005 PMC3422374

[46] Fazeli PL, Woods AJ, Pope CN, Vance DE, Ball KK. Effect of transcranial direct current stimulation combined with cognitive training on cognitive functioning in older adults with HIV: A pilot study. Appl Neuropsychology: Adult. 2019;26(1):36–47. 10.1080/23279095.2017.1357037 PMC5661972 29020472

[47] Vidarsdottir OG, Twamley EW, Roberts DL, Sigurdsson E, Gudmundsdottir B, Magnusdottir BB. Integrative cognitive remediation for early psychosis: A 12-month follow-up. Psychiatry Res. 2020;288:112964. 32361338 10.1016/j.psychres.2020.112964

[48] Smail RC, Brew BJ. Chapter 7 - HIV-associated neurocognitive disorder. In: Brew BJ, editor. Handbook of Clinical Neurology. Volume 152. Elsevier; 2018. pp. 75–97. 10.1016/B978-0-444-63849-6.00007-4 29604986

[49] Blackstone K, Moore DJ, Franklin DR, Clifford DB, Collier AC, Marra CM, et al. Defining neurocognitive impairment in HIV: deficit scores versus clinical ratings. Clin Neuropsychol. 2012;26(6):894–908. 22708483 10.1080/13854046.2012.694479 PMC3848322

[50] Wechsler D. Wechsler Adult Intelligence Scale - Third Edition Manual. San Antonio: The Psychological Corporation; 1997.

[51] Atkins R. Validation of the Center for Epidemiologic Studies Depression Scale in black single mothers. J Nurs Meas. 2014;22(3):511–24. 25608436 10.1891/1061-3749.22.3.511 PMC4389584

[52] Saffer BY, Lanting SC, Koehle MS, Klonsky ED, Iverson GL. Assessing cognitive impairment using PROMIS(^®^) applied cognition-abilities scales in a medical outpatient sample. Psychiatry Res. 2015;226(1):169–72. 25639374 10.1016/j.psychres.2014.12.043

[53] Twamley EW, Vella L, Burton CZ, Heaton RK, Jeste DV. Cognitive Problems and Strategies Assessment [Database record]. APA PsycTests. 2012.

[54] Propheta IVZ. C.J.J. Measuring cognitive emotion regulation in South Africa using the Cognitive Emotion Regulation Questionnaire-short form. Afr J Psychol Assess. 2019;1(0):a9.

[55] Andrade C. The P Value and Statistical Significance: Misunderstandings, Explanations, Challenges, and Alternatives. Indian J Psychol Med. 2019;41(3):210–5. 31142921 10.4103/IJPSYM.IJPSYM_193_19 PMC6532382

[56] Mabaso M, Makola L, Naidoo I, Mlangeni LL, Jooste S, Simbayi L. HIV prevalence in South Africa through gender and racial lenses: results from the 2012 population-based national household survey. Int J Equity Health. 2019;18(1):167. 31666077 10.1186/s12939-019-1055-6 PMC6821038

