# Supplementary material for: Changes in cognitive performance following combined CogSMART-SA and BrainHQ interventions: a pilot study
Source: BMC Neurol. 2026 Mar 21;26:311. doi: 10.1186/s12883-026-04778-9 (PMC13162415; doi:10.1186/s12883-026-04778-9)
Supplement: Supplementary file 1 — Supplementary Material 1. [file 12883_2026_4778_MOESM1_ESM.docx]

**ADDITIONAL FILE 1**

**Table AF1**

*Neuropsychological Test Battery: Raw Scores at Baseline and Study Exit for Intervention and Control Groups (N = 43)*

|  | Control group (n=16) | | Intervention (n = 27) | |
| --- | --- | --- | --- | --- |
| Test | Baseline | Exit | Baseline | Exit |
| **Verbal Memory (Immediate and delay)** |  |  |  |  |
| HVLT Total Recall | 20.88 (5.74) | 23.62 (6.28) | 21.44 (3.97) | 24.85 (3.27) |
| HVLT Delayed Recall | 7.94 (2.05) | 8.00 (2.94) | 7.37 (1.86) | 8.63 (1.74) |
| **Attention** |  |  |  |  |
| Digit Span Total | 11.44 (2.87) | 10.94 (2.46) | 10.56 (2.36) | 10.67 (2.20) |
| **Processing speed** |  |  |  |  |
| Symbol Search | 15.19 (7.28) | 15.62 (7.42) | 14.78 (5.16) | 17.11 (5.29) |
| Coding | 33.38 (13.11) | 36.25 (16.08) | 35.63 (11.51) | 40.19 (11.58) |
| Color Trails I | 84.19 (21.04) | 68.19 (18.53) | 73.52 (20.17) | 70.07 (27.97) |
| **Executive function** |  |  |  |  |
| Color Trails II | 162.56 (55.90) | 163.81 (66.66) | 162.56 (42.46) | 136.63 (34.71) |
| **Verbal Fluency (Language)** |  |  |  |  |
| Semantic Fluency Animals Correct | 12.44 (4.03) | 12.94 (4.02) | 11.52 (3.14) | 11.96 (2.68) |
| Semantic Fluency Fruit/Vegetable Correct | 14.69 (4.24) | 13.62 (3.88) | 14.37 (2.48) | 14.00 (2.77) |
| **Motion Function** |  |  |  |  |
| Grooved Pegboard Test Dominant Hand^a^ | 79.00 (24.28) | 79.44 (19.01) | 83.22 (22.23) | 75.54 (20.10) |
| Grooved Pegboard Test Non-Dominant Hand^a^ | 95.81 (36.53) | 95.38 (27.63) | 97.58 (30.39) | 85.04 (13.32) |

*Note.* Data presented are means and SD or raw scores. HVLT Total Recall = Hopkins Verbal Learning Test Total Recall, HVLT Delayed Recall = Hopkins Verbal Learning Test Delayed Recall, SD = standard deviation. ^a^Data from 26 Intervention participants (1 participant was an outlier).

**Table AF2**

*Neuropsychological Test Battery: Between-group Comparison of Baseline Performance (N = 43)*

|  | **Group** | | | |  |  | |
| --- | --- | --- | --- | --- | --- | --- | --- |
|  | **Control** | | **Intervention** | |  |  | |
| **Cognitive Domain** | (*n* = 16) | | (*n* = 27) | |  |  | |
|  | ***M*** | ***SD*** | ***M*** | ***SD*** | ***t*** | ***p*** | ***ESE*** |
| **Verbal Memory^a,b^ (Immediate and Delayed)** |  |  |  |  |  |  |  |
| HVLT Total Recall (Immediate Verbal Memory) | 20.88 | 5.74 | 21.44 | 3.97 | -0.35 | 0.729 | 0.12 |
| HVLT Delayed Recall (Delayed Verbal Memory) | 7.94 | 2.05 | 7.37 | 1.86 | 0.91 | 0.372 | -0.29 |
| **Attention** |  |  |  |  |  |  |  |
| Digit Span Total | 11.44 | 2.87 | 10.56 | 2.36 | 1.04 | 0.309 | -0.34 |
| **Processing speed^a,b^** |  |  |  |  |  |  |  |
| Symbol Search Total | 15.19 | 7.28 | 14.78 | 5.16 | 0.20 | 0.845 | -0.07 |
| Coding Total | 33.38 | 13.11 | 35.63 | 11.51 | -0.57 | 0.573 | 0.19 |
| Color Trails I Time | 84.19 | 21.04 | 73.52 | 20.17 | 1.63 | 0.113 | -0.52 |
| **Executive Function (Cognitive) Flexibility)^a,b^** |  |  |  |  |  |  |  |
| Color Trails II Time | 162.56 | 55.90 | 162.56 | 42.56 | 0.00 | 1.000 | 0.00 |
| **Verbal Fluency^a,b^ (Language)** |  |  |  |  |  |  |  |
| Semantic Fluency Animals Correct | 12.44 | 4.03 | 11.52 | 3.14 | 0.78 | 0.442 | -0.26 |
| Semantic Fluency Fruit/Vegetable Correct | 14.69 | 4.24 | 14.37 | 2.48 | 0.27 | 0.788 | -0.10 |
| **Motor Function^b,c^** |  |  |  |  |  |  |  |
| Grooved Pegboard Test Dominant Hand Time | 79.00 | 24.28 | 83.22 | 21.83 | -0.57 | 0.572 | 0.19 |
| Grooved Pegboard Test Non-Dominant Hand Time | 95.81 | 36.53 | 105.07 | 49.05 | -0.71 | 0.485 | -0.71 |

*Note.* Data presented are *z*-scores and statistical analyses thereof. ESE = effect size estimate (in this case, Cohen’s *d*).

HVLT Total Recall = Hopkins Verbal Learning Test Total Recall, HVLT Delayed Recall = Hopkins Verbal Learning Test Delayed Recall, SD = standard deviation. ^a^Data from 27 Intervention participants; ^b^Data from 16 Control participants; ^c^Data from 26 Intervention participants (1 participant was an outlier).

**p* < .05. ***p* < .01. ****p* < .001.

AF3

**HIV Social Outcomes Questionnaire**

I’d like to find out if living with HIV [OR, “If living with diabetes / high blood pressure” OR “if your health”] is impacting on your social or work life in any way. I will make some statements and I want you to consider if the statement is true or false for you. Please say YES if the statement is true to your experience over the past TWO WEEKS and NO if it is not.

1. I am going out less often to visit people.
2. I take part in fewer social activities.
3. I am more socially isolated.
4. I am working less because of health-related issues.
5. I am not accomplishing as much as I used to at work.
6. I have become more withdrawn.
7. I act disagreeable to family members, for example, I act spiteful, I am stubborn, I get angry with my family members, I fight with them more frequently.

IF THE PARTICIPANT ANSWERED YES TO ONE OR MORE OF THE ABOVE:

1. I would like to change some of these behaviours.
